# Intensive Telemedicine Transitions of Care Clinic: A Prospective Feasibility Study of a Novel Ambulatory Model Serving Appalachian Patients

**DOI:** 10.13023/jah.0703.07

**Published:** 2025-09-01

**Authors:** Jessica Thayer, Brett Miller, Marcelino Mederos Liriano, Kathryn Hoffman, Gina Baugh, Jenna Sizemore

**Affiliations:** West Virginia University; West Virginia University, School of Medicine; West Virginia University

**Keywords:** Appalachia, interprofessional care, rural health, telemedicine, transitions of care

## Abstract

**Introduction:**

Hospital discharge is a complex process plagued with medical errors and poor coordination. Disjointed discharges are detrimental to Appalachian patients with access barriers and multiple chronic diseases. Telehealth is a tool used to improve access within rural Appalachia. To address this high-risk transition period, an interprofessional team deployed telemedicine to improve post-hospital care for Appalachian patients.

**Purpose:**

Patients with uncontrolled chronic medical conditions were enrolled into the Intensive Telemedicine Transition of Care Clinic (I-TTC) with a primary outcome of 30-day Emergency Department (ED) presentations and hospital readmissions. Secondary outcomes included improved control of chronic conditions and patient cost savings.

**Methods:**

Patients with uncontrolled chronic conditions were given home-monitoring devices and enrolled in the I-TTC post-hospitalization. Telehealth visits were conducted with an interprofessional team comprised of graduate health science students under the supervision of I-TTC physicians. Hospital readmissions, emergency department (ED) presentations, and chronic disease specific measurements were analyzed through retrospective review.

**Results:**

Sixteen adult patients participated in the I-TTC pilot study from 2021–2022. At baseline all patients with hypertension were uncontrolled. The average HbA1C of patients with uncontrolled diabetes was 11%. Post-enrollment, 12.5% of patients had a 30-day ED presentation or hospital re-admission. The average HbA1c for those with uncontrolled diabetes was 8.1% after I-TTC intervention. Of the ten patients with uncontrolled blood pressure, six were controlled post-enrollment. The average cohort total cost savings was $3,144.35.

**Implications:**

The I-TTC suggests feasibility for an interprofessional team utilizing telemedicine in achieving control of chronic medical conditions through improved access to ambulatory healthcare.

## INTRODUCTION

Post-hospitalization care is a growing area of focus given the risk of adverse outcomes toward vulnerable patients.^1^ Transitions of care clinics (TCC) serve as an ambulatory bridge, but studies are limited in terms of optimal visit type and timing.^2^ Access to post-hospitalization care is difficult for rural Appalachian patients who experience higher disease-specific mortality rates and lower access to healthcare professionals.^3^ With studies finding similar readmission rates compared to in-person visits, telemedicine is a practical strategy to increase access to care and reduce travel costs.^4^–^6^

Prior to the time of intervention, the TCC serving a large academic center had limited telemedicine capability requiring most patients to travel for a post-hospitalization appointment. The visit restrictions during the SARS-2-COV pandemic required rapid implementation of virtual appointments. The examined TCC saw increased visit completion rates and an opportunity to provide additional visits to further address chronic condition management post-hospitalization. However, these virtual visits proved challenging due to limitations in clinical data, such as vital signs. At this academic medical center in the Appalachian Region, the authors aimed to evaluate the feasibility of an intensive interdisciplinary transition of care telemedicine clinic (I-TTC) where the study administrators would provide patients with home monitoring devices and multiple virtual visits post-hospitalization. The aim of this study is to evaluate the impact on post-hospitalization care access, acute care usage, and improvement in chronic disease outcomes for patients with insufficient control of at least one chronic medical condition.

## METHODS

Over a one-year period (2021–2022), the authors prospectively enrolled hospitalized adult patients with either uncontrolled hypertension (systolic blood pressure >130 mmHg), diabetes mellitus type 1 or 2 (hemoglobin A1c (HbA1c) >7%), or an admission secondary to either a chronic obstructive pulmonary disease (COPD), congestive heart failure (CHF) or decompensated cirrhosis exacerbation. Additional inclusion criteria included West Virginia (WV) residency with access to either internet-capable devices or a working phone line. We excluded pediatric patients (age <18) and patients discharged to rehabilitation facilities.

Near time of hospital discharge, study team members were notified of potentially eligible patients by inpatient social work and care coordinators. Patients interested were consented to both participation in intensive follow-up visits as well as retrospective data reviews. Prior to discharge, consented participants were enrolled in the study and provided home-monitoring devices as indicated by their condition and need. These included blood pressure cuffs, heart rate monitors, pulse oximeters, scales, or glucometers with glucose monitoring supplies. Patients found to be eligible for the study at time of first post-hospitalization visit in the TCC were also consented and provided devices in person at time of visit. Following enrollment and device obtainment, participants were then provided monthly telemedicine visits in the I-TTC starting at 7 – 14 days post-hospitalization and continued for up to 90 days. These visits were offered as video visits (if participants had a video capable smart device or computer) or as telephone visits, if unable to connect to video. Visits were focused on post-hospitalization care needs, chronic condition symptom management and treatment, and care coordination. An interprofessional team of graduate level health science students, pharmacists, and clinic providers conducted huddles prior to scheduled visits with discussions focused on expected barriers to care and recommended interventions. Recommendations were then discussed with participants by I-TTC providers during the telemedicine visit.

The study team collected demographic information including age, gender, rurality (as defined by rural-urban commuting area codes), primary payor, chronic medical diagnoses, primary care provider engagement, number of I-TTC contacts, 30-day readmission and emergency department (ED) utilization rates, and disease specific outcomes including HbA1c and blood pressure readings.^7^ Cost savings per patient were calculated by adding the price of each device to estimated mileage reimbursement from the patient’s primary address to the I-TTC location. Milage reimbursement was based on the state Medicaid non-emergency medical transportation reimbursement rate at time of study, which was $0.57 per mile traveled.^8^ The authors included all enrolled participants in the retrospective analysis. The study protocol and consenting procedure were approved by the West Virginia University Institutional Review Board (2105308498).

## RESULTS

A total of 16 adult patients (six women, median age 60.5 years) were enrolled **(**Table 1). Fifteen patients received home monitoring devices, and all patients were scheduled for at least two I-TCC visits. The most common diagnosed chronic disease was hypertension, in six participants (37%); five participants had more than one uncontrolled chronic condition diagnosis (31%). Ten participants resided in a rural area (63%).

The average management time by the I-TTC was three months with a median of 9.5 contacts per patient, with twelve patients completing at least one visit. **(**Table 2). The overall visit completion rate was 66%, with similar completion rates between in-person visits (seven completed, 10 scheduled) to virtual visits (24 completed, 37 scheduled). Half of the patients did not have a primary care provider (PCP) at the time of enrollment, but all were scheduled for a PCP visit by study completion. Five of the six patients enrolled with a pre-study diagnosis of diabetes had a pre-enrollment HbA1c within six months of enrollment as well as a matched post-enrollment HbA1c. All but one had a decrease in their HbA1c following participation with an average percentage decrease of 2.6% (n=5). Four additional participants to the six diagnosed with hypertension were noted to have uncontrolled systolic blood pressure at time of enrollment. A total of four remained uncontrolled post-intervention (a decrease from 63% uncontrolled to 38%).

Total average estimated savings were $3,144.35 for the entire cohort and $196.52 per patient. The average amount saved in estimated travel expenses for participants that completed at least one virtual visit was $83.29. The rate of all-cause 30-day ED presentations and hospital readmissions were both 12.5% (n=2). One ED presentation occurred prior to their first I-TTC visit, and another occurred for a newly developed acute concern on follow-up. The patients with documented readmissions were deemed likely unavoidable and not related to chronic disease management.

## IMPLICATIONS

This study suggests that providing patients living in the Appalachian Region with home monitoring devices and multidisciplinary telemedicine visits post-hospitalization may lead to reduced rates of unnecessary acute-care presentations and improved control of diabetes mellitus and hypertension.

As a prospective feasibility study, there were several limitations. The study facilitators were unable to employ a control group given study design, and the small sample study limited reliable statistical testing of outcomes. This study occurred during the height of the SARS-COV-2 pandemic, which negatively impacted patient enrollment and caused telemedicine restrictions, limiting enrollment to WV residents. In addition, patient participation was fragmented, with one patient not participating in any services and three others only receiving home monitoring devices. However, the authors believe that preliminary data from this feasibility study justifies further enrollment to quantify the impacts of the I-TCC intervention, with the long-term goal being to better serve Appalachian patients facing the transition from hospital discharge to home.

**SUMMARY BOX**

**What is already known about this topic?**

Telemedicine is an effective means of patient care that rapidly gained traction following extensive use during the SARS-COV-2 Pandemic. Post-discharge follow-ups are crucial to patient care and are more vital in the setting of rural West Virginia where hospital access is scarce. This study investigates the feasibility of a telemedicine-based clinic for patients following hospital discharge in areas without robust ambulatory access. As this has shown promise, further research evaluating the impact of frequent telemedicine visits should be conducted to improve patient care and better control chronic diseases. The WVU Intensive Telemedicine Transitions of Care Clinic aims to continue enrolling patients and improving health outcomes for the region.

**What is added by this report?**

This report demonstrates a possible intervention for patients recently discharged from the hospital setting, a novel method for improving chronic conditions. With ongoing health provider shortages in Appalachia, this model may serve as an effective way to increase healthcare access and control of chronic conditions.

**What are the implications for future research?**

The discharge period of patients with chronic conditions may be an ideal target for ongoing interventions to improve control of chronic diseases in areas with limited healthcare access.

## Figures and Tables

**Table 1:** Participant Demographics

Participant	Age (Years)	Sex	RUCA Code^*^	Insurance	Inclusion Diagnosis	Devices	PCP?
1	65	M	5	Medicare	HTN, DM	BP, GLM, TH	Yes
2	60	M	1	Medicare	HTN, DM, CHF	BP, GLM, TH, SC, PuOX	Yes
3	66	F	1	Medicare	HTN	BP, TH, SC, PuOX	No
4	65	M	1	Medicare	HTN, CHF, COPD	BP, TH, SC, PuOX	No
5	61	M	1	Privately Insured	HTN	BP, SC	No
6	74	M	1	Medicare	HTN	BP, TH, SC, PuOX	Yes
7	52	M	4	Medicaid	CHF, COPD	BP, SC, PuOX	No
8	26	F	8	Medicaid	DM	N/A	Yes
9	52	F	10	Medicaid	COPD, CHF	BP, GLM, TH, SC, PuOX	Yes
10	60	M	1	Medicaid	DM	BP, GLM	No
11	75	M	4	Medicare	CHF	BP, TH, SC, PuOX	No
12	46	M	8	Medicaid	DM	N/A	No
13	51	F	4	Privately Insured	HTN	BP	No
14	76	F	4	Medicaid	DM	BP	Yes
15	36	M	4	Medicaid	HTN	BP, SC	Yes
16	76	F	4	Medicaid	COPD	SC, PuOX	Yes

NOTES: RUCA: Rural-Urban Commuting Area

PCP: Primary Care Provider

HTN: Hypertension

DM: Diabetes Mellitus

COPD: Chronic Obstructive Pulmonary Disease

BP: Blood pressure/Heart Rate Cuff

GLM: Glucometer and supplies

TH: Thermometer

SC: Scale

PuOx: Pulse Oximeter

**Table 2:** Participant Outcomes

Participants	Pre/Post PCP	Contacts/ scheduled visits, number	Completed Visits, number	Pre/Post HbA1c (%)	Pre/Post BP Control?	30 Day ED/RA	Estimated Cost Savings ($)
1	Y/Y	19/5	5	6/5.1	N/Y	0/0	$230.75
2	Y/Y	7/3	0	11.5/10.5	N/N	0/0	$263.52
3	N/Y	8/1	1	N/A	N/N	0/0	$197.08
4	N/Y	6/2	0	5.4/−	N/Y	0/0	$197.08
5	N/Y	6/4	3	5.3/−	N/Y	0/0	$131.07
6	Y/Y	9/2	2	5.5/−	N/N	0/0	$200.10
7	N/Y	23/5	5	6.2/−	Y/Y	0/0	$659.84
8	Y/Y	11/3	0	10.3/−	N/N	1/0	$0.00
9	Y/Y	22/4	2	6.3/5.9	Y/Y	0/1	$387.78
10	N/Y	12/3	3	14.9/6.3	N/Y	0/0	$131.72
11	N/Y	10/4	2	N/A	Y/Y	0/1	$212.47
12	N/Y	10/2	2	9.6/6.8	Y/Y	1/0	$68.40
13	N/Y	2/2	0	5.8/−	N/Y	0/0	$57.07
14	Y/Y	2/2	2	8.6/8.8	Y/N	0/0	$88.99
15	Y/Y	10/2	2	−/−	N/Y	0/0	$164.87
16	Y/Y	9/2	2	6.3/−	Y/N	0/0	$153.61

NOTES:

I-TTC: Intensive Interdisciplinary Transition of Care Telemedicine Clinic

BP: Blood Pressure

ED: Emergency Department Presentation

RA: Hospital Readmission

Y: Yes, N: No
